# Epidemiology and Predictive Factors for Persistent Breast Pain Following Breast-Conserving Surgery

**DOI:** 10.7759/cureus.14063

**Published:** 2021-03-23

**Authors:** Sherif Monib, Mohamed I Abdelaziz

**Affiliations:** 1 Breast Surgery, St Albans and Watford General Hospitals, West Hertfordshire Hospitals NHS Trust, St Albans, GBR; 2 Surgery, Fayoum University Hospital, Fayoum, EGY

**Keywords:** breast cancer, breast pain, ultrasound scan, fat necrosis, scaring

## Abstract

Background

In general, breast pain is one of the most common causes for referral to breast units; treatment-related breast pain is frequently seen in clinical practice but not well addressed in the literature. While our primary objective was to identify the incidence of persistent breast pain following breast-conserving surgery and possible risk factors, our secondary aim was to assess the possibility of using a breast ultrasound scan to detect parenchymal changes that can contribute to breast pain.

Methods

We have conducted a prospective study including patients who had wide local excision for primary breast cancer treatment between January 2017 and January 2019. Patients’ demographics, including age, BMI, breast volume, and tumour characteristics, were noted. All patients had a clinical assessment and were asked standard questions about their breast pain each visit; they also had an ultrasound scan of the breast and axilla 6 and 12 months after surgery to look for parenchymal changes.

Results

A total of 239 female breast cancer patients were included in our analysis. The mean age was 43.9 years, mean weight was 72.8 kg, mean BMI was 27.4 and mean breast volume was 1173 ml. In total, 38.5% had standard wide local excision, and 61.5% had oncoplastic resection; the mean specimen weight was 74.6 grams. All patients had adjuvant whole breast radiotherapy. We found that patients with younger age, larger breast size, high BMI, oncoplastic resections, and persistent parenchymal changes are associated with an increased incidence of postoperative breast pain while the type of axillary procedure and adjuvant chemotherapy had no significant effect.

Conclusion

Persistent postoperative breast pain was noted in 33% of our patients. We have also indicated that younger patients, patients with larger breast, those with high BMI, with preoperative breast pain, who had oncoplastic resections, and patients with persistent parenchymal changes, as fat necrosis and scarring, are associated more with persistent breast pain.

## Introduction

Breast cancer is the second common cancer in both males and females after lung cancer, accounting for 25% of all cancers. It is the most common cancer in females and considered one of the leading causes of death in females. Its incidence and survival rates vary considerably among different parts of the world but are generally on the rise [1].

Mastectomy has been the standard surgical option for treatment in breast cancer management for decades; with development in medical and surgical sciences, other treatment options have emerged, such as breast conservative surgery and radiotherapy, for early-stage breast cancers. These new treatment options were found to provide comparable mastectomy outcomes but, unfortunately, not without complications [2].

Persistent pain after breast cancer treatment is not an uncommon treatment-related complication, especially after conservative breast surgery. Yet, incidence, risk factors and diagnostic modalities and possible treatments are not explored enough. On some occasions, breast pain may be severe, leading to a considerable decline in physical activity and emotional well-being [3]. Persistent postoperative breast pain was also found to be a strong predictor of a poor health-related quality of life after breast cancer surgery [4,5].

We aimed to identify the incidence of persistent breast pain following breast-conserving surgery and possible risk factors and assess the possibility of using a breast ultrasound scan to detect parenchymal changes that might contribute to breast pain.

## Materials and methods

Study setting

This study was conducted in Fayoum University Hospital in Egypt. We obtained Ethical Committee approval before conducting the study.

Study design

We conducted a prospective study to assess the incidence, risk factors, and possible diagnostic methods of postoperative breast pain following breast-conserving surgery between January 2017 and January 2019. We have included female patients older than 18 years, diagnosed with primary breast cancer and treated with breast-conserving surgery with or without axillary procedures. We have excluded male patients, patients with bilateral breast cancer at presentation, patients who had neoadjuvant systemic treatment for locally advanced or human epidermal growth factor receptor 2 (HER2)-positive disease, and we have also excluded patients with local recurrence, or metastatic disease, those with previous ipsilateral axillary surgery or contralateral previous breast cancer, as well as patients who had previous mastopexy or breast reduction.

Patient factors

Patients’ demographics, including age, BMI, and breast volume measured using Archimedes’ principle of water displacement, were noted [6]. Also, tumour characteristics, as well as histological findings, were recorded. All patients had preoperative triple assessment, including clinical breast examination followed by digital mammogram and bilateral breast and axillary ultrasound scan. Magnetic resonance imaging was carried out for a selective group of patients; staging CT scan was carried out for all patients with involved axillary lymph nodes.

Follow-up

We assessed immediate complications up to 30 days postoperatively and delayed complications up to one year postoperatively. We defined persistent postoperative breast pain as pain present for up to six months postoperatively. All patients were assessed clinically 2 weeks, 6 months, and 12 months after surgery, and they were asked standard questions about their breast pain during the 6- and 12-month clinic visits. We adopted the Cardiff breast pain chart to record the severity of pain, grading it as mild, moderate, or severe [7]. A breast and axillary ultrasound scan was carried out 6 and 12 months after surgery, looking for parenchymal changes.

Statistical methods

Collected data were organised and statistically analysed using SPSS Statistics, version 22 (IBM Corp, Armonk, NY). For quantitative data, means and standard deviations were calculated. Qualitative data collected were presented as numbers and percentages, and the chi-squared test was used as a test of significance. For interpretation of the results, significance was adopted at P<0.05.

## Results

Two hundred thirty-nine female breast cancer patients were included in our analysis; their mean age was 53.9 ± 9.8 years, mean weight 72.8 ± 13.6 kg, mean BMI 27.4 ± 3.7, and mean breast volume was 1173 ± 129 ml.

Tumour characteristics

A total of 36.8% (88) patients presented with right-sided cancers and 63.2% (151 patients) with left-sided cancers. While 90.8% (217 patients) presented with palpable breast lesions, 9.2% (22 patients) had non-palpable lesions, which was only detected on screening mammograms (it is worth mentioning that the breast screening programme is not yet fully established in Egypt). The tumour was located in the upper inner quadrant in 15.5% (37 patients), in the upper outer quadrant in 50.6% (121 patients), in the lower outer quadrant in 15.9% (38 patients), in the lower inner quadrant in 8.4% (20 patients), in the retroareolar region in 8.8% (21 patients), and in the axillary tail in 0.8% (2 patients).

Postoperative histology showed that the mean tumour size was 23 ± 6 mm, and the mean specimen weight was 74.6 ± 16.3 g. A total of 87.4% (209) patients had invasive ductal carcinoma, 6.7% (16 patients) invasive lobular carcinoma, 5.9% (14 patients) had DCIS, and 12.1% (29 patients) had invasive disease as well as ductal carcinoma in situ (DCIS). Invasive tumour grading was Grade I in 7.5% (17/225), Grade II in 50.7% (114/225), and Grade III 41.8% (94/225) patients. DCIS grading was low grade in 30.2% (13/43 patients), intermediate grade in 41.9% (18/43 patients), and high grade in 27.9% (12/43 patients). While 81.2% (194) patients had estrogen and progesterone positive disease, 18.8% (45 patients) were estrogen and progesterone negative, and 11.7% (28 patients) had triple-negative breast cancer (Table 1).

**Table 1:** Demographic findings, and tumour biology of all patients who had breast-conserving surgery followed by radiotherapy in comparison to those who developed persistent breast pain UIQ, upper inner quadrant; UOQ, upper outer quadrant; LOQ, lower outer quadrant; LIQ, lower inner quadrant; IDC, invasive ductal carcinoma; ILC, invasive lobular carcinoma; DCIS, ductal carcinoma in situ; HER2, human epidermal growth factor receptor 2; ER, estrogen receptor; PR, progesterone receptor; LN, lymph node

Patient demographics	All patients (N=239)	Patients with breast pain (N=79)
Age (years)	53.9 ± 9.8	49.7 ± 7.9
Weight (kg)	72.8 ± 16.6	74.1 ± 7.9
BMI	27.4 ± 3.7	28.1 ±​​​​​ 3.9
Mean breast volume (ml)	1173 ± 129	1198 ± 104
Tumour characteristics		
Right side	36.8% (88)	40.5% (32)
Left side	63.2% (151)	59.5% (47)
Location		
UIQ	15.5% (37)	15.2% (12)
UOQ	50.6% (121)	49.4% (39)
LOQ	15.9% (38)	17.7% (14)
LIQ	8.4% (20)	7.6% (6)
Retroareolar	8.8% (21)	10.1% (8)
Axillary tail	0.8% (2)	0%
Tumour size (mm)	23 ± 6	25 ± 3
Tumour type		
IDC	87.4% (209)	77.2% (61)
ILC	6.7% (16)	6.3% (5)
DCIS	5.9% (14)	11.4% (7)
Invasive and DCIS	12.1% (29)	7.6% (6)
Invasive cancer grade		
Grade I	7.5% (17/225)	11.1% (8/72)
Grade II	50.7% (114/225)	45.8% (33/72)
Grade III	41.8 % (94/225)	43.1% (31/72)
DCIS grade		
Low	30.2% (13/43)	23.1% (3/13)
Intermediate	41.9 (18/43)	46.2% (6/13)
High	27.9 (12/43)	30.7% (4/13)
Hormone receptor status		
ER-PR positive	81.2% (194)	77.2% (61)
ER-PR negative	18.8% (45)	10.1% (8)
HER2 positive	0%	0%
HER2 negative	100% (all patients)	100% (all patients)
Triple negative	11.7% (28)	12.7% (10)
Axillary LN involvement		
Not applicable (DCIS)	5.8% (14)	8.9% (7)
Involved	49.4% (118)	53.2% (42)
Not involved	44.8% (107)	37.9% (30)

Treatment

A total of 38.5% (92) patients had standard wide local excision and 61.5% (147) had oncoplastic resections; 57.7% (138 patients) had SLNB, and 42.3% (101 patients) had level I-II ALNC. The mean hospital stay was two days. All patients had adjuvant whole breast radiotherapy; 81.2% (194) had adjuvant endocrine treatment (selective estrogen receptor modulators [SERMs] or aromatase inhibitors), and 46.9% (112 patients) had chemotherapy (Table 2).

**Table 2:** Treatment modalities of all patients who had breast-conserving surgery followed by radiotherapy in comparison to those who developed persistent breast pain WLE, wide local excision; SLNB, sentinel lymph node biopsy; ALNC, axillary lymph node clearance

Treatment	All patients, % (N)	Patients with breast pain, % (N)
Surgical treatment		
Standard WLE	38.5% (92)	41.8% (33)
Oncoplastic resection	61.5% (147)	58.2% (46)
Specimen weight (g)	74.6 ± 16.3	79.3 ± 13.1
Axillary procedure		
No axillary procedure	5.8% (14)	8.8% (7)
SLNB	51.1% (122)	43.1% (34)
ALNC	43.1% (103)	48.1% (38)
Adjuvant treatment		
Chemotherapy	46.9% (112)	53.2% (42)
Targeted treatment	0%	0%
Radiotherapy	100% (all patients)	100% (all patients)
Endocrine treatment	81.2% (194)	77.2% (61)

Follow-up

Immediate complications included haematoma in 3.3% (8) patients, seroma in 7.9% (19), and wound infection in 2.9% (7) whereas delayed complications included altered sensation of the medial aspect of upper arm in 9.6% (23 patients), arm lymphoedema in 3.7% (9), and breast lymphoedema in 4.6% (11) patients.

Breast pain assessment in all patients showed that most of the patients who devolved breast pain had focal, constant pain of moderate intensity, which lasted up to 12 months postoperatively (Table 3).

**Table 3:** Breast pain assessment

	At 6 months, % (N)		At 12 months, % (N)	
Side	Ipsilateral	Contralateral	Ipsilateral	Contralateral
Focal	64.6% (51/79)	0% (0/79)	29.1% (23/79)	0% (0/79)
Diffuse	35.4% (28/79)	10.1% (8/79)	16.5% (13/79)	11.4% (9/79)
Mild	34.2% (27/79)	7.6% (6/79)	13.9% (11/79)	7.6% (6/79)
Moderate	56.9% (45/79)	2.5% (2/79)	30.4% (24/79)	3.8% (3/79)
Severe	8.9% (7/79)	0% (0/79)	1.3% (1/79)	0% (0/79)
Cyclical	44.3% (35/79)	6.3% (5/79)	21.5% (17/79)	6.3% (5/79)
Constant	55.7% (44/79)	3.8% (3/79)	24.1% (19/79)	5.1% (4/79)

A total of 33% (79) patients were found to have persistent postoperative breast pain at 6 months, which slightly decreased at 12 months. Analysis of the possible risk factors of persistent postoperative breast pain showed that younger patients, patients with larger breast, those with high BMI, with preoperative breast pain, and patients who had oncoplastic resections were associated more with more persistent breast pain. Adjuvant chemotherapy and the type of axillary procedure were not associated with a significant impact on breast pain (Table 4).

**Table 4:** Analysis of the possible risk factors in the 79 patients who were found to have persistent breast pain SLNB, sentinel lymph node biopsy; ALNC, axillary lymph node clearance *Statistically significant. **Not significant.

Risk factor	Variable	% (N)	P value
Age	<50 years	60.8% (48/79)	0.007*
	>50 years	39.2% (31/79)	
Breast volume	<500 ml	5.4% (28/79)	0.02*
	>500 ml	64.6% (51/79)	
BMI	<25	41.8% (33/79)	0.04*
	>25	58.2% (46/79)	
Preoperative breast pain	Present	68.4% (54/79)	0.03*
	Absent	31.6% (25/79)	
Breast surgery	Standard wide local excision	41.8% (33/79)	0.07**
	Oncoplastic resection	58.2% (46/79)	
Axillary surgery	No axillary procedure^1^	8.8% (7/79)	^1-2^0.005*, ^2-3^0.08**, ^1-3^0.003*
	SLNB^2^	43.1% (34/79)	
	ALNC^3^	48.1% (38/79)	
Radiotherapy	All patients had radiotherapy		
Chemotherapy	Chemotherapy	53.2% (42/79)	0.06**
	No chemotherapy	46.8% (37/79)	
Parenchymal changes	Present	67.1% (53/79)	0.04*
	Absent	32.9% (26/79)	

Bilateral breast ultrasound scans of all patients revealed increased postoperative parenchymal changes, which slightly decreased after 12 months (Table 5).

**Table 5:** Ipsilateral ultrasound scan findings in all patients (with or without breast pain) *Statistically significant. **Not significant.

	At 6 months	At 12 months	P value
Seroma	7.9% (19/239)	2.9% (5/239)	0.03*
Fat necrosis	33.9% (81/239)	23.4% (56/239)	0.06**
Breast lymphedema	4.6% (11/239)	2.9% (7/239)	0.04*

Breast ultrasound scans at 6 and 12 months showed significant parenchymal findings in patients who suffered from persistent breast pain (Table 6).

**Table 6:** Ipsilateral ultrasound scan findings in patients with persistent breast pain *Statistically significant. **Not significant.

	At 6 months	At 12 months	P value
Seroma	5.1% (4/79)	3.8% (3/79)	0.1*
Fat necrosis	34.1% (27/79)	29.1% (23/79)	0.09**
Breast lymphedema	8.9% (7/79)	6.3% (5/79)	0.07**

## Discussion

The prevalence of breast pain in the general population ranges from 41% to 69%, with a higher prevalence observed in older women, women with large breast size, and those who are less fit and active. Also, breast pain negatively impacted 35% of patients affecting quality of life, sex, and sleep [8]. Persistent breast pain following breast cancer-related treatment, especially wide local excision and radiotherapy, is not uncommon. Li and Kong reported that 28.5% of their patients experienced persistent pain after surgery [9]. Fukui et al. mentioned that prevalence rates for persistent pain following breast cancer surgery can be seen in up to 60% of cases [10]. In our cohort, 33% of patients were found to have persistent postoperative breast pain; intensity of breast pain was mild in 34.2%, moderate in 56.9%, severe in 8.9% after six months, and 13.9%, 30.4%, and 1.3%, respectively, after 12 months.

Bokhari et al. found that younger patients (younger than 50 years) experienced persistent postoperative breast pain more than older patients [11], in comparison to higher prevalence in older women in the general population [8]. Our cohort found that persistent postoperative breast pain is seen more in patients younger than 50 years of age compared to those older than 50 years.

High BMI and underlying depression or anxiety were also associated with an increased risk of chronic postoperative breast pain [12]. In our cohort, we found that persistent postoperative breast pain is seen more in patients with BMI higher than 25 compared to those with BMI less than 25. Unfortunately, the relation between breast volume and postoperative breast pain was not discussed enough in the literature. In our cohort, we have noticed that patients with large breast size (>500 ml) are more likely to develop persistent postoperative breast pain when compared to patients with smaller breasts (<500 ml).

McCann et al. noted an association between preoperative and postoperative breast pain as 28% of their patients had breast pain in the affected breast before breast cancer surgery [13]. Langford et al. found that pre-existing preoperative breast pain can increase the risk of acute postoperative pain and lead to low physical well-being, psychological symptoms, sleep disturbance, and inferior quality of life [14]. In our cohort, 68.4% of patients who developed persistent postoperative breast pain experienced preoperative breast pain.

Breast-conserving surgery oncoplastic techniques provide adequate resection margins with maintained cosmesis. Yet, our cohort showed an association between oncoplastic resections and increased incidence of persistent breast pain as 58.2% of patients who had oncoplastic resections experienced persistent breast pain. In comparison, 41.8% of patients who had standard wide local excision experienced persistent breast pain.

Parenchymal changes following breast-conserving oncoplastic surgery are attributed to extensive breast tissue mobilisation and can always contribute to persistent postoperative breast pain. Nakada et al. created grading criteria for fat necrosis and noted that 4.6% of their patients who had oncoplastic resections were found to have fat necrosis [15]. We have identified much higher levels of parenchymal changes, which were found to be associated with an increased incidence of persistent postoperative breast pain.

Spivey et al. found the pain burden index (PBI) six months postoperatively to be significantly higher in patients who had axillary lymph node clearance than those who had sentinel lymph node biopsy; they also noted that surgery duration did not impact pain [16]. The UK Standardization of Breast Radiotherapy (START) trial showed that 20% of patients who had hypofractionation and 30% of the patients who had conventional fractionation of adjuvant radiotherapy for breast cancer experienced breast and arm pain at five years of follow-up [17]. In our cohort, all patients had adjuvant whole breast radiotherapy; therefore, the association between postoperative breast pain and radiotherapy was not assessed.

Postoperative breast pain in patients who had adjuvant chemotherapy can be attributed to induce neuropathy. Taxanes, platinum agents, and vinca alkaloids are among the most likely medications to cause post-treatment pain [18]. Our cohort did not find a significant difference between patients who had adjuvant chemotherapy and those who didn’t. Although it is well known that patients on aromatase inhibitors experience stiffness, aches, and bony pains. Yet, there is no association in the literature between adjuvant endocrine treatment and breast pain.

Breast sonoelastography is a relatively new method involving ultrasound to assess breast tissue stiffness and elasticity; Dzoic et al. found that women with persistent breast pain had higher glandular elastographic values when compared to women without breast pain [19]. Taking into consideration that in our cohort, we have found persistent breast parenchymal changes detected by ultrasound scans to be directly related to persistent breast pain, we believe that an ultrasound scan is an excellent objective modality to assess persistent parenchymal breast changes related to postoperative pain and a helpful modality to rule out postoperative pathology and recurrence, which can alleviate anxiety and reduce pain severity by reassuring patients that there is no suspicious pathology.

Different modalities have been demonstrated in the literature, aiming to treat persistent postoperative pain. Medical options include non-steroidal anti-inflammatory drugs [20], opioids and gabapentin or pregabalin for neuropathic pain [21], and tricyclic antidepressant medication [22]. Non-medical options include myofascial massage and psychological therapy [23]. Minimally invasive intervention options include truncal regional anaesthesia, including paravertebral block or proximal intercostal block [24], and pulsed radiofrequency stimulus [25].

Limitations

Unfortunately, we didn’t have the resources for radioactive-directed sentinel lymph node biopsy, so we have used the patent blue dye directed technique only. We didn’t do an ultrasound scan before starting radiotherapy to assess parenchymal changes as we focused only on persistent changes rather than early postoperative changes. We also did not evaluate breast density to ascertain its relation to persistent postoperative breast pain. There might have been an analysis bias when comparing outcomes due to risk factors and breast cancer treatment overlap.

## Conclusions

Persistent postoperative breast pain was noted in 33% of our patients. We also found that younger age patients, patients with larger breast size, those with higher BMI, and patients with preoperative breast pain and oncoplastic resections were associated more with more persistent breast pain. The absence of axillary breast surgery was associated with less breast pain, and adjuvant chemotherapy and the type of axillary procedure were not associated with a significant impact on breast pain.

We also found parenchymal changes as fat necrosis and scarring to be directly related to persistent postoperative breast pain. We recommend larger case series analysis, including more patients and longer follow-ups to develop more robust data, to improve patient outcomes.

## References

[1] Ghoncheh M, Pournamdar Z, Salehiniya H (2016). Incidence and mortality and epidemiology of breast cancer in the world. Asian Pac J Cancer Prev.

[2] Meek AG (1998). Breast radiotherapy and lymphoedema. Cancer.

[3] Miaskowski C, Cooper B, Paul SM (2012). Identification of patient subgroups and risk factors for persistent breast pain following breast cancer surgery. J Pain.

[4] Rietman JS, Dijkstra PU, Debreczeni R, Geertzen JH, Robinson DP, de Vries J (2004). Impairments, disabilities and health related quality of life after treatment for breast cancer: a follow-up study 2.7 years after surgery. Disabil Rehabil.

[5] Ho PJ, Gernaat SA, Hartman M, Verkooijen HM (2018). Health-related quality of life in Asian patients with breast cancer: a systematic review. BMJ Open.

[6] Bouman FG (1970). Volumetric measurement of the human breast and breast tissue before and during mammaplasty. Br J Plast Surg.

[7] Mansel RE, Webster DJT, Sweetland HM (2009). Breast pain and nodularity. In Hughes, Mansel & Webster's Benign Disorders and Diseases of the Breast (Third Edition).

[8] Scurr J, Hedger W, Morris P, Brown N (2014). The prevalence, severity, and impact of breast pain in the general population. Breast J.

[9] Li YY, Kong SK (2011). Persistent pain after breast cancer surgery in a Chinese population. Clin J Pain.

[10] Fukui JA, Meister M, Pagano I, Bantum E (2020). Incidence of breast pain in breast cancer patients in a multi-ethnic cohort. J Clin Oncol.

[11] Bokhari FN, McMillan DE, Susan McClement RN, Daeninck PJ (2012). Pilot study of a survey to identify the prevalence of and risk factors for chronic neuropathic pain following breast cancer surgery. Oncol Nurs Forum.

[12] Urits I, Lavin C, Patel M (2020). Chronic pain following cosmetic breast surgery: a comprehensive review. Pain Ther.

[13] McCann B, Miaskowski C, KoettersT KoettersT (2012). Associations between pro-and anti-inflammatory cytokine genes and breast pain in women prior to breast cancer surgery. J Pain.

[14] Langford DJ, Schmidt B, Levine JD (2015). Preoperative breast pain predicts persistent breast pain and disability after breast cancer surgery. J Pain Symptom Manage.

[15] Nakada H, Inoue M, Furuya K (2019). Fat necrosis after breast-conserving oncoplastic surgery. Breast Cancer.

[16] Spivey TL, Gutowski ED, Zinboonyahgoon N (2018). Chronic pain after breast surgery: a prospective observational study. Ann Surg Oncol.

[17] Hopwood P, Haviland JS, Sumo G, Mills J, Bliss JM, Yarnold JR (2010). Comparison of patient-reported breast, arm, and shoulder symptoms and body image after radiotherapy for early breast cancer: 5-year follow-up in the randomised Standardisation of Breast Radiotherapy (START) trials. Lancet Oncol.

[18] Jung BF, Herrmann D, Griggs J, Oaklander AL, Dworkin RH (2005). Neuropathic pain associated with non-surgical treatment of breast cancer. Pain.

[19] Dzoic Dominkovic M, Ivanac G, Bojanic K (2020). Exploring association of breast pain, pregnancy, and body mass index with breast tissue elasticity in healthy women: glandular and fat differences. Diagnostics.

[20] Chopra K, Kokosis G, Slavin B, Williams E, Dellon AL (2019). Painful complications after cosmetic surgery: management of peripheral nerve injury. Aesthet Surg J.

[21] Broyles J, Tuffaha S, Williams E, Glickman L, George T, Dellon AL (2016). Pain after breast surgery: etiology, diagnosis, and definitive management. Microsurgery.

[22] Larsson IM, Ahm Sørensen J, Bille C (2017). The post-mastectomy pain syndrome - a systematic review of the treatment modalities. Breast J.

[23] Massingill J, Jorgensen C, Dolata J, Sehgal AR (2018). Myofascial massage for chronic pain and decreased upper extremity mobility after breast cancer surgery. Int J Ther Massage Bodywork.

[24] Zinboonyahgoon N, Vlassakov K, Lirk P (2019). Benefit of regional anaesthesia on postoperative pain following mastectomy: the influence of catastrophising. Br J Anaesth.

[25] Kim HT, Kim KY, Kim YD, Moon HS (2010). Pulsed radiofrequency lesioning for treatment of chronic breast neuropathic pain after breast reduction - a case report. Korean J Anesthesiol.

